# Delayed Leukoencephalopathy and Foreign Body Reaction After Endovascular Treatment in Patients With Intracranial Aneurysms and Aneurysmal Subarachnoid Hemorrhage—A Systematic Review of the Literature

**DOI:** 10.3389/fsurg.2021.732603

**Published:** 2021-12-23

**Authors:** Sami Ridwan, Jörg Andreas Kandyba, Anita Schug, Elina Malsagov, Nikolaos Karageorgos, Franz-Josef Hans

**Affiliations:** ^1^Department of Neurosurgery, Paracelsus-Klinik Osnabrueck, Osnabrueck, Germany; ^2^Department of Interventional Neuroradiology, Marien-Hospital Osnabrueck, Osnabrueck, Germany

**Keywords:** aneurysm, subarachnoid hemorrhage – SAH, endovascular treatment (EVT), delayed leukoencephalopathy, foreign body reaction in brain

## Abstract

**Background:** Delayed leukoencephalopathy and foreign body reaction are rare complications after endovascular treatment of intracranial aneurysms. However, cases are increasingly being described, given the rising case numbers and complexity.

**Methods:** Clinical presentation, differentials, diagnostics, treatment, and formerly published data were reviewed in light of available cases. A systematic search of the literature was performed according to the Preferred Reporting Items for Systematic Reviews and Meta-Analyses statement.

**Results:** This article provides an extensive literature review of previously described cases, and discusses the causes and management of this rare and delayed complication by referring to 17 articles on this topic, with a total of 50 cases with sufficient data in the literature. Furthermore, we present the case of a 53-year-old female patient with subarachnoid hemorrhage from a large anterior communicating artery aneurysm with tortuous cervical vessels who was treated with endovascular coiling and has suffered delayed leukoencephalopathy 6 weeks after discharge. Diagnostics, treatment, and clinical course of this rare complication are presented on this case and based on formerly published literature. The patient timely recovered under high dose corticosteroid treatment and follow up MRI showed almost complete remission of the described lesions within 10 days in accordance with previously published data.

**Conclusion:** Foreign body reaction might result in delayed leukoencephalopathy, especially following complex endovascular aneurysm treatment. Early high dose followed by low dose ongoing corticosteroid treatment might result in timely remission.

## Introduction

Endovascular treatment of intracranial vascular pathologies is a valuable intervention added to our armamentarium in the last two decades (1, 2). Since then, sizeable upgrowth has been achieved in this field. Treatment of intracranial aneurysms had, until then, been solely a neurosurgical procedure. Today, corresponding cases are discussed in interdisciplinary conferences. Treatment decisions for either surgical clipping or endovascular coiling are made based on solid data (3–6). In addition to conventional coils, many other endovascular implants have been developed and have found their way into daily practice.

Subarachnoid hemorrhage (SAH), aneurysmal subarachnoid hemorrhage in particular (aSAH), is a devastating cerebrovascular emergency. As a disease, aSAH is widely addressed in the literature. Mainly focusing on cerebral vasospasm (VS), predictors of outcome and the health economic impact, given the fairly young age of affected patients (7–19). Poor grade aSAH cases, which are not eligible for surgical treatment, might be treated endovascularly today. This offers treatment options for cases which, otherwise, would not have been treated in the past, e.g., poor grade cases with posterior circulation aneurysms or patients not suitable for microsurgical approach given the poor clinical presentation (20). Patients with ruptured intracranial aneurysms are also discussed in interdisciplinary conferences and are treated based on reliable data (3).

Endovascular procedures are becoming a widely available treatment option, and not only exclusive to university medical centers and maximum care facilities. Not until recently, we have learned about rare but serious complications following endovascular procedures other than common hemorrhages, hemorrhagic infarction, or ischemic lesions. Specifically, delayed complications which might even occur after patients' discharge, such as coil migration, foreign body reaction, delayed leukoencephalopathy, and delayed hemorrhage, have moved into focus (21–26). Recent literature offers only a few scattered case reports and one fairly large systematic retrospective case series highlighting this matter (21). In this article, a literature review regarding delayed leukoencephalopathy (DLE) and foreign body reaction (FBR) after corresponding procedures is performed. A recent case of a patient, with aneurysmal subarachnoid hemorrhage undergoing endovascular treatment (ET) with subsequent DLE at the authors' institution, is briefly described to offer recent imaging and treatment data.

## Methods

A thorough systematic MEDLINE/PubMed database search of the English literature was performed for all articles before April 2, 2020 using the following keyword algorithm: “endovascular” combined with “leukoencephalopathy” or “foreign body reaction,” also considering publications of any earlier date. The search was performed according to the Preferred Reporting Items for Systematic Reviews and Meta-Analyses statement (PRISMA) (27). The results were then properly screened for relevance regarding the topic by a neurosurgeon consultant and an interventional neuroradiologist consultant separately. Review articles and article references were screened for further articles with possible relevance to this matter and were also cross-referenced. Main inclusion criterium was onset of delayed leukoencephalopathy and/or foreign body reaction after endovascular procedures. On closer analysis, focus was laid upon corresponding procedures in patients with intracranial aneurysms with or without suffering aSAH. Demographics, neurological symptoms on admission and at time of discharge, along with imaging findings, time to onset, type of endovascular treatment and applied materials, and possible detection of a foreign body or allergic reaction were extracted from the corresponding studies when available.

All procedures involving human participants were in accordance with the ethical standards of the institutional and/or national research committee and with the 1964 Helsinki Declaration, and its later amendments or comparable ethical standards. The patient's written consent was obtained for diagnostics and for treatment procedures including anonymous scientific use of apprehended imaging and clinical data. Specific informed consent for this case study was not required because the data was obtained from routine procedures and was retrospectively analyzed according to the general treatment consent signed on admission. For this type of systematic review, formal ethics committee consent is not required.

A recent case of aSAH undergoing ET for an anterior communicating artery (AcomA) aneurysm was closely analyzed after requiring readmission for progressive neurological symptoms. This case description offered an opportunity to display recent, original, and high-quality representative imaging and clinical data with sufficient follow-up for such complication and was, therefore, briefly presented as complimentary case to the reviewed literature.

## Results

**Figure 1:**
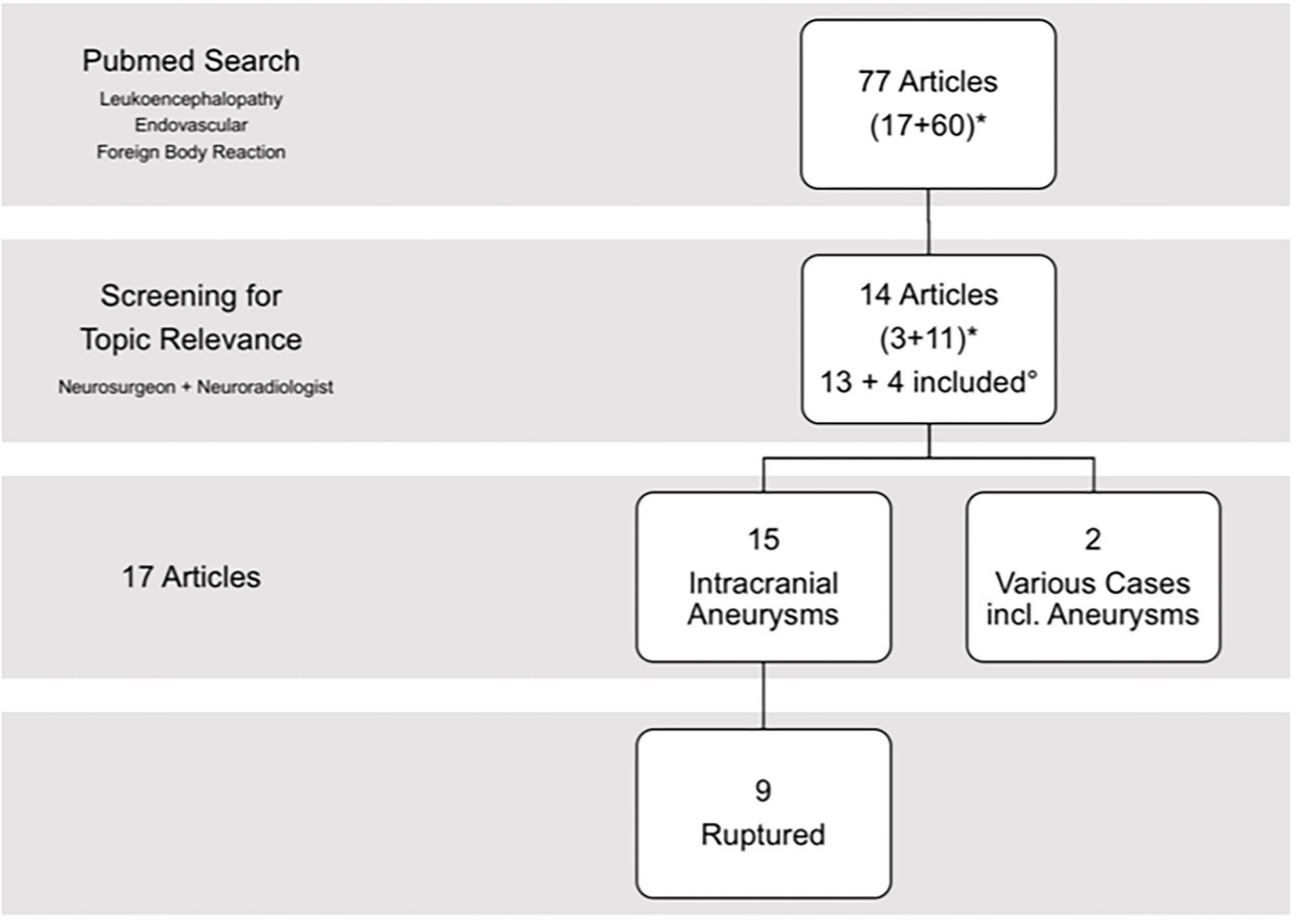
Article selection. *Search results for keyword combination “leukoencephalopathy” and “endovascular” or “foreign body reaction.” °After excluding duplicates from the 14 articles identified as relevant, 13 remained for further review. Four articles are added after cross-referencing. In total, 17 articles are included of which 9 reported ruptured aneurysms.

The MEDLINE/PubMed database search for the keyword combination “endovascular” and “leukoencephalopathy” revealed 17 entries, while the search for “foreign body reaction” and “endovascular” revealed 60 entries. After proper analysis of these articles, 3 and 11 articles were identified as relevant to this topic, respectively. After exclusion of duplicates [1 article present in both search results (21); 13 articles] and adding cross-referenced articles that were not found by primary MEDLINE/Pubmed search (4 articles), a total of 17 articles remained for further review and data analysis (21–24, 28–40). In detail, 15 studies included intracranial aneurysms, 9 of which analyzed (at least in part) ruptured aneurysm cases (21, 24, 29, 30, 32, 35–37, 39) (Figure 1). Two of the articles included a heterogenic cohort of various endovascularly treated pathologies in addition to intracranial aneurysms (33, 37). Demographics and available clinical data on admission are shown in Table 1. Studies included in this review are presented in detail in Table 2. An extensive statistical analysis was not eligible in this context, as single cases could not be adequately compared.

**Table 1:** Demographic data.

**References**	**Pts**.	**Age** **Yrs**.	**Gender** **M/F/D**	**HH** **Grade**	**WFNS** **Grade**
Ikemura et al. (21)	16	59 (51–66.5)	F 13/16 (81.3%) M 3/16 (18.8%)	–	–
Nakagawa et al. (22)	7	59 (39-76)	F 7/7 (100%)	NA	NA
Park et al. (23)	2	64 52	F 2/2 (100%)	NA	NA
Nakamizo et al. (24)	1	41	F 1/1 (100%)	–	–
Fukushima and Nakahara (28)	1	56	F 1/1 (100%)	NA	NA
Huijgen et al. (29)	1	36	F 1/1 (100%)	1	–
Hollesli et al. (30)	1	65	F 1/1 (100%)	–	–
Giordan et al. (31)	1	64	F 1/1 (100%)	NA	NA
Shotar et al. (32)	2	54 45	F 1/2 (50%) M 1/2 (50%)	NA –	NA –
Mehta and Mehta (33) (RA)	12^*^ (32)	NA	NA	NA	NA
Lorentzen et al. (34)	1	52	F 1/1 (100%)	NA	NA
Minks et al. (35) (LE)	1	51	F 1/1 (100%)	–	–
Cruz et al. (36)	7	54 (32-71)	F 6/7 (85.7%) M 1/7 (14.3%)	–	–
Fealey et al. (37)	1^‡^ (3)	58	F 1/1 (100%)	–	–
Ulus et al. (38) (LE)	1	41	F 1/1 (100%)	NA	NA
Skolarus et al. (39)	2	46 56	F 2/2 (100%)	NA 3	NA –
Shapiro et al. (40)	5	–	–	NA	NA
This study (2020)	1	53	F 1/1 (100%)	1	1

*Age data is displayed as published in the corresponding studies and only for patients included in this review. Most patients suffering DLE after endovascular treatment were females. The data regarding clinical presentation on admission for patients with ruptured aneurysms were only available in very few cases*.

*NA, not applicable; —, not available*.

* *Data from the review article was not added, as relevant cases from this article were separately presented*.

‡ *One of the three cases in this article has presented with delayed symptoms after endovascular treatment of an intracranial aneurysm*.

**Table 2:** Cases with delayed leukoencephalopathy following endovascular aneurysm treatment from the reviewed literature.

**References**	**Pts**.	**Ruptured/Unruptured**	**Aneurysm treatment**	**Time to symptoms (Days)**	**Symptoms**	**Imaging findings**	**Management**	**Permanent deficit**	**Predictors/Assumed etiology**	***P*-Value**
Ikemura et al. (21)	16	R/UR	C	Median 71.5 (30-101)	7 headache and hemiparesis 9 asymptomatic	Delayed leukoenecephalopathy	Steroids/FRS None	No	>180 ml contrast >1 Microcatheter >99.5 min fluoroscopy	0.008 0.009 0.06
Nakagawa et al. (22)	7	UR	5 C 2 C+S	Mean 28 (18-46)	3 convulsions 2 hemiplegia 1 hemianopia 1 asymptomatic	Delayed inflammatory changes	Steroid pulse Followed by Oral medication	No	Allergic reaction Metal allergy	–
Park et al. (23)	2	UR	C+S	21 18	1 facial palsy 1 hemiparesis	Multifocal white matter lesions	Steroid pulse f/b Oral steroids	No	Nickel-associated	–
Nakamizo et al. (24)	1	R	C	21	asymptomatic	White matter changes with enhancement	Steroids	No	FBR to hydrophyilic coating	–
Fukushima and Nakahara (28)	1	UR	C	14	Headache Hemiparesis	Diffuse leukoencephalopathy	Prednisolone	No	Allergic reaction to coil coating material	–
Huijgen et al. (29)	1	R	C	21	Headache Hemianopia Hemiparesis	Cortical and subcortical vasogenic edema and enhancement	Dexamethasone	No	RPLS	–
Hollesli et al. (30)	1	R (warning leak)		150	Headache	Hyperintense white matter changes, vasogenic edema	None	No	FBR	–
Giordan et al. (31)	1	UR	FD	60	Headache Aphasia	Nodular enhancement	Oral steroids	No	FBR	–
Shotar et al. (32)	2	R UR	C FD	28 510	Headache, hypoesthesia Seizure	NICE	Methylprednisolone Prednisone	No	FBR	–
Mehta and Mehta (33) (RA)	12^*^ (32)	NN	NN	14–270	Headaches, neurologic deficits	Persistent abscesses, granulomas, multifocal polymer emboli	Steroids NN	Yes	Polymer reaction^†^	–
Lorentzen et al. (34)	1	UR	S	90	Aphasia, hemiparesis, ataxia	Vasogenic edema, patchy CE	Methylprednisolone Prednisone, AZT	No	FBR^†^	–
Minks et al. (35) (LE)	1	R	C+S	14	Seizures, headache	Multiple lesions with CE	Antibiotics	No	FBR	–
Cruz et al. (36)	7	R/UR	4 C 1 C+S 2 FD	Median 63 (43-118)	1 Paresthesia, SLA 1 Gait disturbances 1 Hemiparesis, aphasia 4 Asymptomatic	Multiple enhancing lesions	3 None 2 AB w/o Steroids 1 AB 1 Antiepileptic drug	No	FBR	–
Fealey et al. (37)	1^‡^ (3)	R	C	270	Hemiparesis, seizure	Three ring enhancing lesions	Antibiotics	NN	FBR^†^	–
Ulus et al. (38) (LE)	1	UR	C+S	30	Headache, visual flashing lights	Multiple hyperintense lesions, vasogenic edema	None	No	Hypersensitivity reaction	–
Skolarus et al. (39)	2	UR R	1 C 1 C+S	30	Scintillating scotoma Gait instability	Bilateral white matter changes, punctate CE	None	No	PGLA coil related inflammatory reaction	–
Shapiro et al. (40)	5	UR	4 C 1 C+S	14–56	Headache, hemiparesis, neck pain, hemianopia, paresthesia, SLA	Subcortical lesions with CE, hematoma, vasogenic edema	Steroids, AB	No	Foreign body emboli^†^	–
This study (2020)	1	R	C	42	Headache, hemiparesis, aphasia	Multiple hyperintense lesions, vasogenic edema	Dexamethasone	No	FBR	–

*Studies included in the review process in order of citation. Seventeen articles were finally reviewed*.

*AB, Antibiotics; AZT, Azathioprine; C, Coiling; CE, Contrast enhancement; f/b, followed by; FBR, Foreign body reaction; FD, Flow diversion; FRS, Free radical scavenger; LE, Letter to the editor; NN, No specific data available; PGLA, Polyglycolic-Polylactic Acid Coils; Pts, Number of patients; R, Ruptured; RA, Review article; RPLS, Reversible posterior leukoencephalopathy syndrome; S, Stent; SLA, Seizure-like activity; UR, Unruptured*.

* *Review article including 32 cases, only 12 with delayed symptoms*.

† *Histological findings described*.

‡ *One of the three cases in this article presented with delayed symptoms after endovascular treatment of an intracranial aneurysm*.

### Results of the Literature Review

Sixty-two cases with delayed leukoencephalopathy after endovascular procedures for intracranial aneurysms had been described in the literature. Data presented in a review article by Mehta and Mehta (33) did not provide sufficient information for each corresponding case to be properly included in this analysis. Of the 32 cases in the corresponding review article, only 12 had delayed symptoms and could be considered. Previously published DLE cases within the literature review performed by Mehta and Mehta (33) were included as original studies and were primarily identified by our search algorithm. After proper exclusion of duplicates and of cases with insufficient data, a total of 50 case presentations offering detailed case characteristics, diagnostic, and treatment regimens were analyzed and are presented in Table 2 in addition to assumed underlying etiology and available histological findings as formerly published.

#### Demographics and Clinical Presentation

Excluding one missing article with corresponding patient information, demographic data revealed a median age of all included cases at 54 years (Range 36–65; average age 53 ± 8.16). Two articles have offered only median age data. Gender information were only available for 46 cases, revealing the majority of DLE cases being female (41/46; 89.1%; vs. males 5/46; 10.9%). Clinical data on admission regarding severity of aSAH in terms of the Hunt and Hess grade (HH) and also the World Federation of Neurosurgical Societies (WFNS) grade were only available in 3 cases as shown in Table 1.

Fifteen patients (15/50; 30%) were asymptomatic at time of DLE diagnosis. In symptomatic cases, symptoms varied from simple headaches to severe neurologic deficits such as hemiparesis, aphasia, and visual or gait disturbances. Seizures or seizure-like activity were described in 8 patients.

#### Diagnostics and Treatment Courses

The time between the endovascular procedure and the onset of symptoms or incidental imaging findings of DLE varied between 14 and 510 days (2 weeks and 17 months). MRI findings were mostly described as vasogenic edema with multiple subcortical and cortical enhancing lesions mainly in the corresponding ipsilateral hemisphere.

Most patients received a steroid regimen, including Dexamethasone, Prednisone, or Methylprednisolone or a sequence of different steroids. However, dose and duration varied largely even within the same publication with regimens from steroid pulse IV treatment for a few days, IV followed by oral or simply oral steroid medication with dose reduction over time. Treatment duration varied between a few days and several months up to a year. Scattered cases received antibiotic treatment with or without steroids. Symptomatic patients recovered almost fully after treatment, a few cases even without specific treatment. However, lesions might be present up to 521 days and may reoccur or progress in the meantime.

#### Possible Predictors of DLE and Allergy Testing

Statistical analyses of possible DLE predictors had not been published until this year. In 16 closely investigated cases in a large retrospective cohort study by Ikemura et al., various predictors of DLE had been described for the first time in a statistical analysis (21). Mainly, use of high doses of contrast (>180 ml; *p* = 0.008), use of more than 1 microcatheter (*p* = 0.009), and longer fluoroscopy times (>99.5 min; *p* = 0.006) appear to facilitate occurrence of DLE. Interestingly, number of coils, use of bioactive coils (polyglycolic/polylactic), number of microguide wires, and of guiding systems had no significant predictive value. Histological data with verification of foreign body granuloma, partly containing materials used in ET, after brain biopsy of DLE lesions, were scarce (34, 37, 40) and were only available for scattered cases as shown in Table 2.

Allergy tests were performed in 5 of the reviewed studies and were reactive to either metal or materials used in endovascular procedures in 9/28 (32%) total and in 9/19 (47%) tested cases (21–23, 28, 32). In the study cohort of Ikemura et al., the data were not available for 9/16 (56%) patients with regards to allergy testing. Three out of sixteen were negatively tested and 4 cases displayed metal allergies (21).

#### Authors' Case Presentation

**Figure 2:**
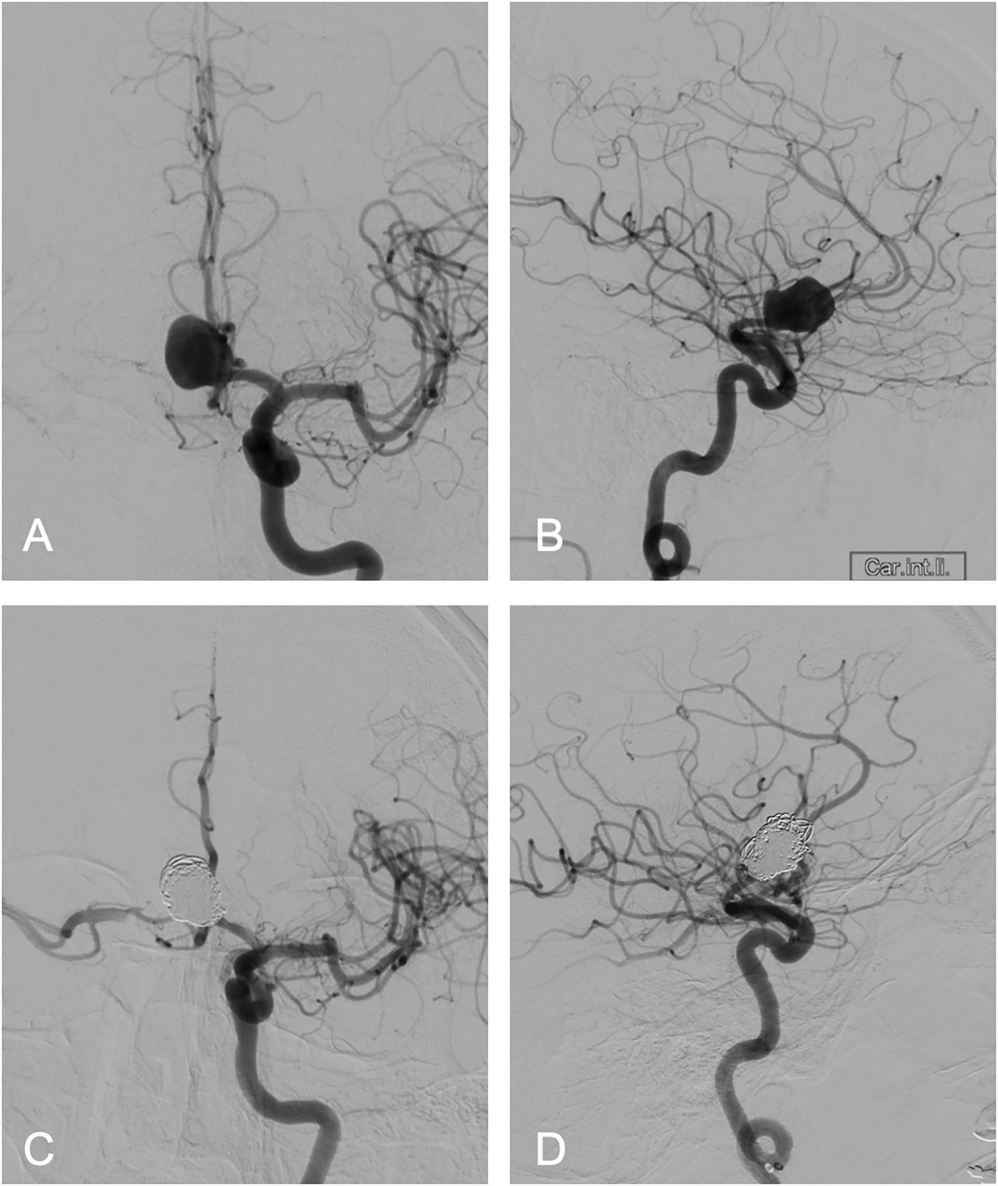
Conventional angiography. Conventional angiography imaging before **(A,B)** and after endovascular coiling treatment **(C,D)**. The lateral planes **(B,D)** clearly depicting the described tight cervical internal carotid artery (ICA) loop.

A 53-year-old female patient with no known allergies suffered a subarachnoid hemorrhage HH grade 1, WFNS grade 1, and Fisher grade 3, from a large anterior communicating artery aneurysm (17 mm; with a 7 mm neck diameter). Stable clinical condition on admission with a Glasgow Coma Scale (GCS) of 15 and a mild headache with a score of 4/10 on the Visual Analog Scale (VAS). Conventional angiography with endovascular treatment was performed within 24 h. Pre- and post-intervention conventional angiography imaging are presented in Figure 2.

**Figure 3:**
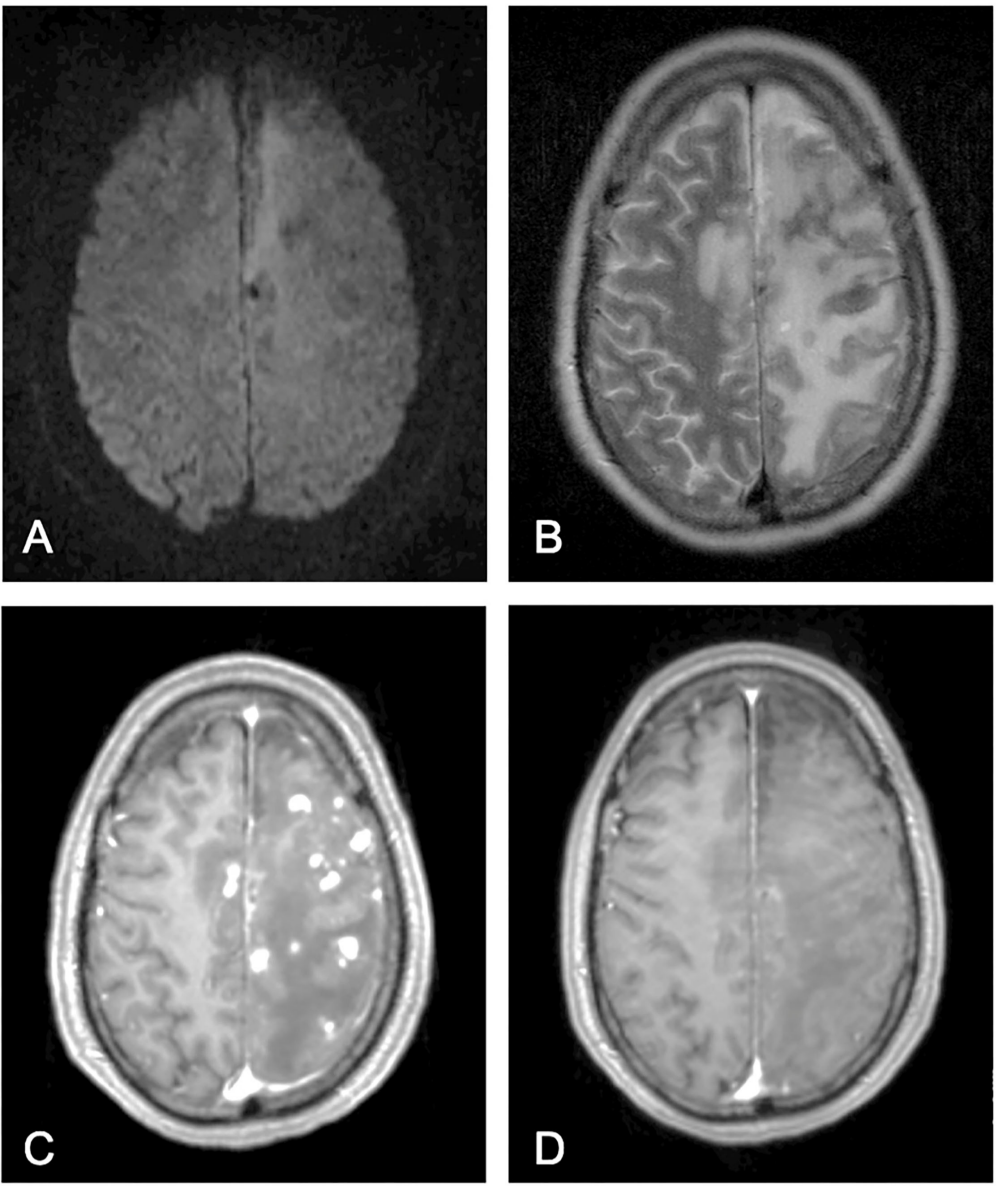
Magnetic resonance imaging. Magnetic resonance imaging (MRI) findings at time of re-admission 6 weeks after initial discharge [**(A)** Diffusion-weighted axial imaging, **(B)** T2 weighted axial imaging and **(C)** contrast enhanced T1 axial imaging] and 10 days later after corticosteroid treatment [**(D)** contrast enhanced T1 axial imaging].

The endovascular procedure was time consuming due to tortuous cervical vessels. The tight cervical ICA loop was passed with a distal access catheter, but the initially planned balloon-assisted coiling was not possible. The aneurysm was therefore treated using a double microcatheter technique and was initially framed using a 15 mm × 40 cm coil, then, was subsequently occluded using further 8 coils. Two finishing coils could not be sufficiently placed and had to be removed. A thrombus forming at the broad aneurysm neck despite heparin infusion and migrating into the A2 segment could not be accessed again as secondary to the tortuous vessel configuration, resulting in partial anterior cerebral artery ischemia and with some scattered ischemic micro-emboli in the left central cortex. No vascular obliteration was documented during angiography after proper aneurysm occlusion. The patient was put on Aspirin (100 mg daily; long-term) and on therapeutic Enoxaparin (until discharge). This intervention-related complication resulted in mild hemiparesis and mild aphasia. Cerebral vasospasm (VS) could not be detected during acute in-hospital treatment in the ICU, and further on the normal ward based on the clinical course and CT angiography. At discharge, most symptoms were already resolved. The patient was transferred to a rehabilitation facility with GCS 15 and modified Rankin Scale (mRS) 1. Readmission was necessary due to clinical deterioration 6 weeks after transfer to rehabilitation. Imaging revealed extensive vasogenic edema with small contrast enhancing, predominantly, cortical lesions in the left ICA territory including anterior cerebral artery (ACA) and middle cerebral artery (MCA) territory and no additional novel vascular obliteration as shown in Figure 3.

**Figure 4:**
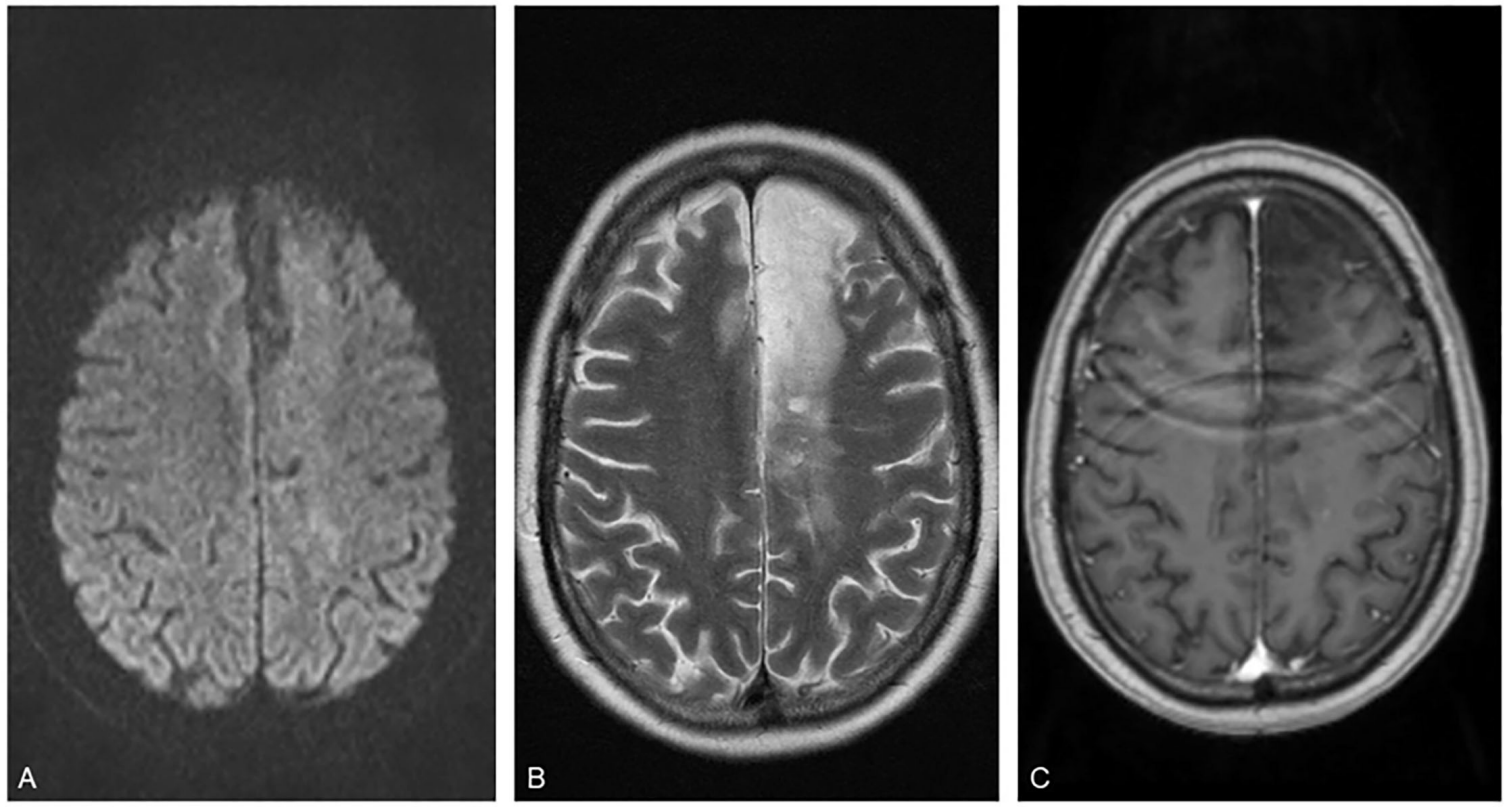
Last follow up. Follow up magnetic resonance imaging (MRI) findings 3 months after latest discharge [**(A)** Diffusion-weighted imaging, **(B)** T2 weighted imaging, and **(C)** contrast enhanced T1 imaging].

Septic emboli were initially considered as major differential but were ruled out based on laboratory results and imaging. Initial antibiotic treatment was terminated. High dose corticosteroid treatment was initiated on admission with a single intravenous Dexamethasone infusion of 40 mg, followed by 8 mg oral Dexamethasone for three times daily. The follow up MRI 10 days later showed an almost complete remission of the described lesions. Corticosteroids were reduced to a maintenance dose of 2 mg for three times daily for 3 months to follow. The patient was discharged within 14 days in GCS 15 and mRS 1. Regular follow up appointments were set up to monitor possible steroid side effects. Follow up MRI 3 months after discharge revealed no residual contrast-enhancing lesions and a resolved edema (Figure 4) with no residual neurological deficit. Dexamethasone treatment was subsequently concluded and a regular follow up was scheduled after another 6 months.

## Discussion

Delayed complications after endovascular procedures in intracranial aneurysms, specifically in patients with aSAH, are only found in scattered case reports and small case series, lacking the opportunity to perform a proper statistical analysis in order to identify possible predictors, as shown by the reviewed literature (Table 2). In this study, we closely analyzed all eligible cases in the literature in addition to a recent illustrative case treated in our department. The data included mostly of single-case descriptions without the opportunity to perform a proper statistical analysis. Hence, the data in this study is presented as a systematic review of the literature. Additionally, the cases were analyzed in a qualitative matter to offer the reader a better and detailed overview of symptoms, diagnostics, and treatment of such rare complication.

Subcortical and cortical lesions, in conjunction with vasogenic edema and other complications appearing shortly after endovascular procedures in general, had previously been described in the literature (41–45). Many of which were referred either to mechanical emboli due to the procedure itself, to septic emboli, or to a foreign body reaction. Delayed complications after endovascular aneurysm treatment seem to appear on our radar more frequently today. Probably, as a consequence of increasing complexity in endovascular procedures over the last two decades.

### Identifying the Underlying Cause

In Germany, patients undergoing endovascular procedures are generally scheduled for routine follow up imaging. This allows broad detection of such complications. However, detection of a complication and identification of the underlying cause pose different challenges. Proper diagnostics are widely available. Distinctly describing the underlying mechanism remains the main challenge (34, 37, 40). Today, routine MRI with diffusion weighted, contrast enhanced and angiography imaging, digital subtraction angiography, blood, and CSF analyses, and, in some cases, even brain biopsy with histological diagnosis, offer considerable diagnostic options. Some kind of foreign body, inflammatory or hypersensitivity allergic reaction to metals, such as Nickel or other materials used in ET, is suggested as the most common underlying mechanism of DLE in the literature (22, 23, 32, 38). Solely, Ikemura et al. offered a statistical analysis of further factors additionally facilitating occurrence of DLE, such as contrast volume, number of used microcatheters, and mean fluoroscopy time. However, they failed to correlate coil types, number of coils, and guidewires with DLE (21). The lesion distribution involving the complete ipsilateral ICA territory of our case would however point to material originating at the level of the distal access catheter (DAC; either from the inner coating of the DAC or the microcatheters/balloon catheter within the DAC) as a likely cause, especially since passage of the microcatheters and the balloon catheter of the cervical ICA loop proved difficult and could explain excessive friction at this point. Only the material from the inner coating of the microcatheters or the coils would be expected to rather show an ACA-related distribution. A prospective multicenter analysis appears implicitly required to answer the numerous unanswered questions to this matter. However, this might portray a substantial challenge given the low incidence of DLE.

### Clinical Course, Treatment, and Follow Up

Almost one-third of the described cases did not reveal any symptoms despite having distinct imaging findings (21, 22, 24, 36). Clinical symptoms might lead to earlier detection. However, most patients undergoing endovascular procedures for intracranial aneurysms have received routine follow up imaging. Symptoms are often related to the affected area of vascularization and ranged from mild headaches and SLA to severe deficits, e.g., hemiparesis and aphasia (21–23, 28–32, 34–40). A correlation between number of detected lesions or edema size (e.g., volumetric analysis), and severity of symptoms had not been described and might be of relevance to further assess cases requiring treatment. Given the benign clinical course in the majority of cases, to treat or not to treat remains the main question. Only 5 out of 17 studies (5/18 including our case) have reported cases not being treated (21, 30, 36, 38, 39). This might be due to lack of symptoms in the corresponding patients. It appears that most investigators tend to treat. Some cases might be treated with antibiotics given that septic emboli represent the most plausible differential (35–37, 40). However, most published cases had no evidence of an infection in the corresponding blood, imaging, and cerebrospinal fluid (CSF) analyses. The most common treatment described is a steroid regimen (21–24, 28, 29, 31, 32, 34, 36, 40). There is no evidence in favor of any of the earlier listed steroid products regarding the treatment of DLE. However, improvement is observed to the extent of full clinical and imaging-proven recovery. This might be explained by two mechanisms: edema reduction and additionally decreased inflammation. Both are underlined by improvement in imaging findings after treatment.

Follow up is essentially required as presented by several studies, with lesions visible in MRI up to 521 days after intervention (21). Furthermore, in some cases, recurring symptoms and deterioration in imaging findings had been described (32). In our opinion, preliminary follow up in 2–3 months might suffice. Longer and repeated follow up periods might be required in some cases. However, patients must be made aware of such complications and must seek timely hospital treatment for novel, deteriorating, or recurring symptoms to allow proper management.

### Role of Allergy Tests

Since most researchers describe or assume some kind of foreign body reaction, in part with histological proof, a possible role of allergy testing prior to endovascular procedures should be addressed. Data regarding allergy tests in the presented cases is very limited and allows no proper conclusion as only 5 studies reported testing a total of 19 patients (21–23, 28, 32). Even in the largest, up-to-date case series by Ikemura et al., data were only available for 7 patients (21). It, however, has to be considered that a foreign body reaction to sheared material during ET does not have to be an allergic reaction *per se*. It might simply be a granulomatous foreign body reaction to sheared foreign objects irrespective of the underlying material itself. Under these circumstances, metal allergy testing might not add any relevant knowledge or predictive value regarding occurrence of DLE.

### Summary

Delayed Leukoencephalopathy (DLE) is a rare, serious, but, in most cases, treatable delayed complication of endovascular treatment of brain aneurysms. It mostly affects middle-aged female patients. Lesions might occur within 17 months after treatment and can remain visible up to 521 days. Three pathologies are mainly suspected to result in DLE: foreign body reaction to sheared materials, infection, and allergic reactions. Within the reviewed literature, FBR is the primarily agreed on-trigger of DLE. Distinguishing DLE from other differentials, such as infections, is mainly based on MRI with corresponding contrast and diffusion-weighted imaging. Treatment is simply based on a long-term corticosteroid regimen with risk of reoccurrence. Surgical treatment or a brain biopsy are only required in very few cases not responding to treatment. Allergic reactions seem to play no part or only a minor role in the development of DLE.

**Figure 5:**
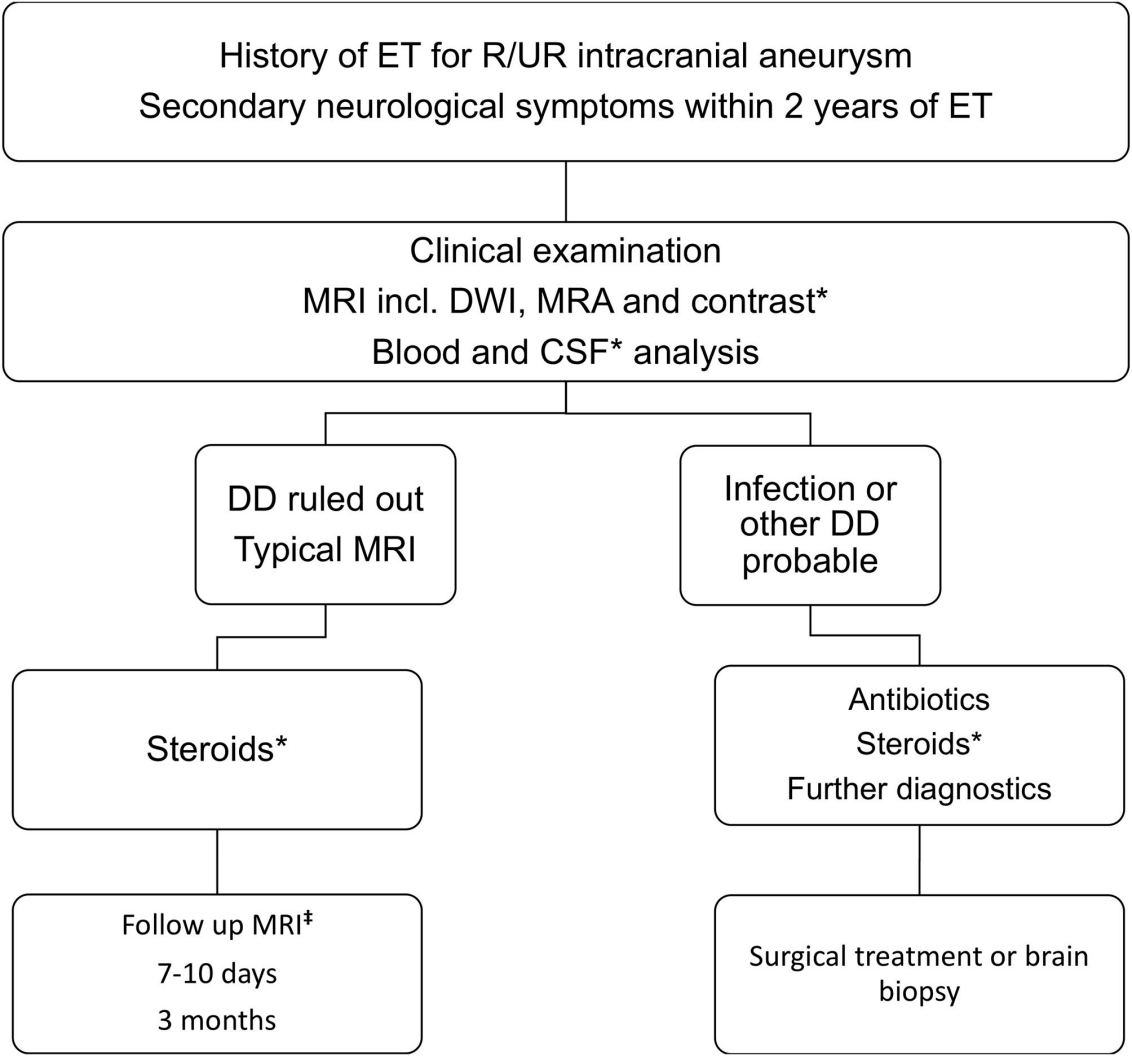
Diagnostic and treatment algorithm. The authors summarize findings of the reviewed cases in form of an algorithm to properly identify and timely treat patients with DLE after endovascular treatment of intracranial aneurysms. *Further diagnostic and treatment measures are dependent on native radiological findings, radiological indication, known allergies, and pharmacological interactions. ^‡^Follow up MRI imaging intervals should be individually adapted to case characteristics.

Based on data extracted from this review article, the authors summarize these in a diagnostic and treatment algorithm for suspected DLE cases (Figure 5). Eligible for this algorithm should be patients with history of endovascular treatment for intracranial aneurysms, either ruptured or unruptured presenting with secondary onset of neurological symptoms. After clinical examination and proper investigation of the patient's history, emergency MRI, including MRA, DWI, and contrast-enhanced imaging, should be performed to assess possible underlying pathologies. If MRI is not available, CT scan with CT angiography should be performed and the further available diagnostics should be discussed. Blood and CSF laboratory results are required to rule out infection. CSF samples should be obtained after proper imaging and ruling out contraindications. After excluding infections and other competing differentials, findings can be identified as DLE, when meeting the above-mentioned imaging criteria in accordance with the literature. Our treatment regimen included a high dose Dexamethasone course followed by low dose treatment for 3 months. Monitoring of possible steroid-related complications is obligatory. Follow up imaging should be performed 3–6 months after onset of DLE. However, there is no solid evidence defining the follow up timeline or range.

### Limitations

The low number of cases discussed in this review article poses the main limitation. However, this article includes a recent review of the literature with additional cross-referenced articles and, thus, offers an updated and comprehensive presentation of a fairly large collection of formerly published cases. Not all listed studies have reported detailed case information. This resulted in the exclusion of the corresponding data. Similarly, treatment regimens were not always as precisely described. Publication bias might be of relevance, as asymptomatic cases are less likely to be reported. Metal allergy testing and histology were only available in limited cases. This review of formerly published cases included a recent case treated at the authors' institution, thus, offering the opportunity to properly display a detailed analysis of such rare complication within the margins of the PRISMA statement (27).

## Conclusion

Delayed leukoencephalopathy (DLE) after endovascular treatment for intracranial aneurysms poses a serious but treatable complication. The most common cause is some kind of foreign body reaction to materials used in such procedures. Steroids offer a feasible treatment option. However, a distinct treatment regimen is yet to be defined. Surgical verification via biopsy might be applied in atypical cases not properly responding to treatment. We summarize findings in a diagnostic and treatment algorithm to identify and timely treat DLE cases.

## Data Availability Statement

The original contributions presented in the study are included in the article/supplementary material, further inquiries can be directed to the corresponding authors.

## Author Contributions

SR, JK, and FJH devised the project, the main conceptual ideas, and proof outline. SR and FJH supervised and finalized the manuscript. SR and JK performed an independent literature search. SR carried out data acquisition, worked out almost all of the technical details, performed the statistical calculations, and drafted the manuscript assisted by AS and EM. NK assisted with manuscript completion and the submitted figures. All authors contributed to the article and approved the submitted version.

## Conflict of Interest

SR is a consultant for Brainlab AG outside this work. The remaining authors declare that the research was conducted in the absence of any commercial or financial relationships that could be construed as a potential conflict of interest.

## Publisher's Note

All claims expressed in this article are solely those of the authors and do not necessarily represent those of their affiliated organizations, or those of the publisher, the editors and the reviewers. Any product that may be evaluated in this article, or claim that may be made by its manufacturer, is not guaranteed or endorsed by the publisher.
